# Alterations in neutrophil mRNA profiles in multiple sclerosis and identification of candidate genes for further investigation

**DOI:** 10.3389/fneur.2025.1548196

**Published:** 2025-02-17

**Authors:** Huining Zhang, Ruoyi Guo, Yusen Han, Zhichao Yao, Moyuan Quan, Bin Li, Li Guo

**Affiliations:** ^1^Department of Neurology, The Second Hospital of Hebei Medical University, Shijiazhuang, China; ^2^Key Laboratory of Clinical Neurology, Hebei Medical University, Ministry of Education, Shijiazhuang, China; ^3^Neurological Laboratory of Hebei Province, Shijiazhuang, China

**Keywords:** multiple sclerosis, neutrophils, neutrophil extracellular traps, neutrophil transcriptomics, biomarker

## Abstract

**Introduction:**

Multiple sclerosis (MS) is a chronic and debilitating inflammatory disease of the central nervous system (CNS), characterized by demyelination and neurodegeneration. Emerging evidence implicates neutrophils in MS pathogenesis, particularly through processes like neutrophil extracellular traps (NETs) formation and degranulation, which may exacerbate inflammation and autoimmunity.

**Methods:**

RNA sequencing of peripheral blood neutrophils from MS patients and healthy controls identified differentially expressed genes (DEGs). Pathway enrichment and protein–protein interaction (PPI) analyses highlighted potential biomarkers, validated using reverse transcription quantitative PCR (RT-qPCR) and enzyme-linked immunosorbent assay (ELISA).

**Results:**

Our analysis identified 1,968 DEGs in neutrophils from MS patients, comprising 1,068 upregulated and 900 downregulated genes. Pathway enrichment analysis revealed significant involvement of immune processes, including antigen presentation, B and T cell receptor signaling, intracellular signaling cascades, and neutrophil degranulation. Notably, KEGG analysis highlighted a pivotal role for upregulated genes in neutrophil extracellular traps (NETs) formation, a process increasingly associated with autoimmunity. PPI network analysis pinpointed five key hub genes—*LCN2*, *LTF*, *ELANE*, *CAMP*, and *CTSG*—as central players in neutrophil-mediated immune modulation. Protein-level validation using ELISA confirmed elevated levels of *LCN2*, *ELANE*, *CAMP*, and *CTSG*, consistent with transcriptomic findings, further supporting their role as biomarkers. Subsequent RT-qPCR validation demonstrated robust diagnostic potential for these genes, with area under the curve (AUC) values of 0.952 (LCN2), 0.827 (LTF), 0.968 (ELANE), 0.950 (CAMP), and 0.862 (CTSG).

**Discussion:**

These findings uncover a previously underappreciated role for neutrophils in MS pathogenesis, driven by alterations in gene expression linked to immune modulation and NET formation. The identified biomarkers, particularly ELANE and LCN2, demonstrate strong diagnostic potential, offering a new avenue for non-invasive MS diagnostics. Beyond clinical utility, this study highlights the importance of neutrophil-driven immune responses in MS, providing mechanistic insights into the complex interplay between innate and adaptive immunity in demyelinating diseases. Furthermore, these findings suggest that targeting neutrophil-specific processes, such as NETs formation and degranulation, could mitigate inflammatory damage and provide novel therapeutic approaches for MS treatment. These results lay the groundwork for future studies exploring therapeutic strategies targeting neutrophil functions in MS.

## 1 Introduction

Multiple sclerosis (MS) is a chronic inflammatory demyelinating disease of the central nervous system (CNS) that affects more than 2.8 million individuals globally (1). The disease predominantly targets regions such as the periventricular areas, juxtacortical regions, optic nerves, spinal cord, brainstem, and cerebellum (2). MS is characterized by the spatial and temporal dissemination of lesions, exhibiting pathological features that include inflammatory infiltration, demyelinating plaques, and axonal damage (3, 4). Recent years have witnessed a notable increase in the incidence and prevalence of MS. A recent epidemiological study involving hospitalized patients in China reported an incidence rate of 0.235 cases per 100,000 person-years, with a male-to-female ratio of 1:2.02 among diagnosed adult patients (5). Clinically, MS presents with a spectrum of debilitating symptoms, including limb weakness, sensory disturbances, visual impairment, ataxia, and bladder dysfunction, all of which significantly compromise patients’ quality of life (6, 7). Additionally, a substantial number of patients experience depression and face challenges in adapting to social circumstances. Given these complexities, there is an urgent imperative to delve into the pathogenesis of MS, aiming to establish a more robust foundation for its treatment strategies.

The precise etiology of MS remains elusive, emerging from a complex interplay of genetic susceptibility, environmental influences, and dysregulated immune responses (8). While the contributions of adaptive immune cells, particularly T and B lymphocytes, have been the focus of extensive investigation, recent studies have illuminated the critical role of innate immune cells, particularly neutrophils, in the pathogenesis of this disease (9, 10). This shift in understanding underscores the need to explore the multifaceted immune landscape of MS, as innate mechanisms may offer novel insights into disease progression and potential therapeutic targets.

Neutrophils, the most abundant type of leukocyte, have long been recognized for their role in acute inflammatory responses. However, accumulating evidence reveals that these cells are also pivotal players in the chronic inflammatory milieu characteristic of MS (11, 12). In the peripheral blood of MS patients, neutrophils exhibit an enhanced activation phenotype (13), and the neutrophil-to-lymphocyte ratio (NLR) has emerged as a valuable biomarker for assessing disease activity (14, 15, 83). Our preliminary investigations, which analyzed peripheral blood laboratory data from 65 MS patients and 70 healthy volunteers, corroborate findings from prior studies (16). Notably, elevated levels of neutrophil-associated factors, such as neutrophil elastase (NE), CXCL1, and CXCL5 in plasma, correlate with MS lesion burden and clinical disability (17). Furthermore, myeloperoxidase (MPO), another product of neutrophils, is found to be significantly elevated in the serum of MS patients (18). Neutrophil extracellular traps (NETs), initially described by Brinkmann et al. in 2004, are extracellular web-like structures composed of DNA, histones, and antimicrobial proteins that play a pivotal role in immune defense by capturing and neutralizing pathogens (19–21). Moreover, MPO bound to DNA—a hallmark of NETs—is also elevated in these patients, underscoring the multifaceted contributions of neutrophils to MS pathogenesis (13). The diverse functions of neutrophils, including the release of inflammatory cytokines, various proteases, reactive oxygen species (ROS), and their role in disrupting the blood–brain barrier, as well as antigen presentation and T cell activation, collectively highlight their significant impact on disease progression (22).

In this study, we undertook a comprehensive analysis of the mRNA expression profiles of neutrophils isolated from the peripheral blood of MS patients. This investigation was designed to assess functional alterations in neutrophil activity and to identify potential pathogenic mRNAs and biomarkers associated with the disease. Our findings aim to illuminate the molecular underpinnings of MS, providing a foundation for future research and therapeutic development.

## 2 Materials and methods

### 2.1 Patients recruitment and sample collection

Patients diagnosed with MS and seeking care at the Department of Neurology, the Second Hospital of Hebei Medical University, were recruited for this study between October 2020 and July 2023. All patients met the following inclusion criteria: (1) Patients conformed to the McDonald diagnostic criteria; (2) Patients were in the acute phase of exacerbation and had not received any immunosuppressive or disease-modifying drug treatment within 1 month prior to the current exacerbation; (3) No concurrent autoimmune disease or infection. Age-matched healthy volunteers were recruited from the physical examination center of our hospital as the healthy control group (HC). All participants were randomly divided into the RNA-seq cohort and the validation cohort. Informed consent was obtained from all participants prior to inclusion. The research was conducted in compliance with the ethical standards outlined in the Declaration of Helsinki and was approved by the Ethics Committee of the Second Hospital of Hebei Medical University on 24 March 2022 (2022-R264).

Peripheral blood was collected from all participants using two types of collection tubes. For neutrophil isolation, samples were collected using EDTA anticoagulant tubes, with cellular isolation conducted within 1 h after collection. Neutrophils were isolated via density gradient centrifugation using Human Peripheral Blood Neutrophil Isolation Kit (LZS11131, TBD, Tianjin, China) according to the manufacturer’s instructions. The isolated neutrophils were immediately resuspended in TRIzol reagent and quickly frozen in liquid nitrogen for subsequent analyses. For serum preparation, blood was collected in serum separation tubes containing a clot activator. Samples were left at room temperature for 30 min to ensure complete clot formation and then centrifuged at 3,000 rpm for 10 min at 4°C. The resulting serum was carefully aspirated, aliquoted into sterile microcentrifuge tubes, and stored at −80°C for subsequent analyses.

### 2.2 RNA-sequencing

A total RNA quantity of ≥1 μg was employed, and poly(A)-tailed mRNA was selectively enriched using the NEBNext Ultra II RNA Library Prep Kit for Illumina (New England Biolabs Inc., Ipswich, Massachusetts, USA) in conjunction with Oligo(dT) magnetic beads. Following enrichment, the mRNA was subjected to fragmentation via divalent cations. This fragmented mRNA served as a template for complementary DNA (cDNA) synthesis, utilizing random oligonucleotide primers. The resulting double-stranded cDNA was purified, then underwent end repair, the addition of an ‘A’ base to the 3′ end, and ligation of sequencing adapters. AMPure XP beads facilitated the selection of cDNA fragments approximately 400–500 bp in length, which were subsequently amplified by PCR and purified once more using AMPure XP beads. Following RNA extraction, purification, and library construction, second-generation sequencing (Next-Generation Sequencing, NGS) was conducted on these libraries using the Illumina platform with paired-end (PE) sequencing. Differentially expressed genes (DEGs) were identified based on the criteria of |Fold Change| ≥ 1.5 and a *p*-value <0.05.

### 2.3 RNA extraction, reverse transcription, and quantitative reverse transcription polymerase chain reaction (RT-qPCR)

Several differentially expressed RNAs (DE RNAs) were selected for validation through RT-qPCR based on specific criteria: (1) the top five most significant RNAs or those exhibiting the largest |Fold Change| were prioritized; (2) RNAs detectable in each sample were included; and (3) RNAs with established associations with autoimmunity were given preference.

Total RNA was extracted from the samples of validation cohort using the chloroform-isopropanol-ethanol method. cDNA was synthesized via reverse transcription using the SweScript All-in-One RT SuperMix for qPCR (G3337, Servicebio, Wuhan, China). PCR amplification was performed with the 2 × Universal Blue SYBR Green qPCR Master Mix (G3326, Servicebio, Wuhan, China) under the following conditions: an initial 10 min at 95°C, followed by 40 cycles of 15 s at 95°C, 1 min at 60°C, and a final extension of 10 min at 72°C. The relative mRNA expression levels were calculated using the 2^−ΔΔCt^ method.

### 2.4 Functional enrichment analysis

Gene Ontology (GO) and Kyoto Encyclopedia of Genes and Genomes (KEGG) pathway analyses were conducted using the online platform provided by OE Biotech Co., Ltd.^1^ Genes were classified into three principal GO categories: biological process, cellular component, and molecular function. GO enrichment analysis employed the hypergeometric distribution method to calculate *p*-values, with significance defined as *p* < 0.05. This analysis identified GO terms that were significantly enriched among the DEGs, elucidating their primary biological functions.

For KEGG pathway enrichment analysis, pathways exhibiting significant enrichment (*p* < 0.05) were identified and assessed for the concentration of DEGs within various biological pathways. This approach provides insights into the potential roles these genes play in the pathogenesis of the disease.

### 2.5 Protein–protein interaction (PPI) network analysis

To investigate the interactions of upregulated genes associated with neutrophil degranulation, we utilized the STRING database^2^ for protein–protein interaction (PPI) network analysis of the DEGs. We established a high-confidence score threshold of >0.7 to enhance the reliability of the identified interactions. The results from the STRING analysis were then visualized and further analyzed using Cytoscape software. The MCODE plugin facilitated the clustering of DEGs, allowing us to identify subnetworks with an MCODE score of ≥5, which were subsequently annotated for detailed examination. This comprehensive approach elucidates the potential functional networks involved in neutrophil degranulation within the context of disease pathology.

### 2.6 Induction and differentiation of neutrophil-like cells (differentiated HL-60, dHL-60)

To validate the expression of the five selected differential genes in neutrophils, we induced the human promyelocytic leukemia cell line HL-60 to differentiate into neutrophil-like cells (dHL-60) using dimethyl sulfoxide (DMSO). HL-60 cells RRID: CVCL_0002 (ZQ0433, Zhongqiao Xinzhou, Shanghai, China) were cultured in Iscove’s Modified Dulbecco’s Medium (IMDM) (BL312A, Biosharp, Hefei, China) supplemented with 10% fetal bovine serum (ST30-3302, PAN, Adenbach, Germany) and 1% penicillin/streptomycin (15,140,122, Thermo Fisher, Waltham, MA, USA), maintained at 37°C in a 5% CO₂ atmosphere. Cells in the logarithmic growth phase were selected and induced to differentiate by adding 1.25% DMSO (D2650, Sigma-Aldrich, Lyon, France) for a duration of 3 days, with media remaining unchanged throughout the induction period. This methodology provides a reliable model for studying gene expression pertinent to neutrophil function and differentiation.

### 2.7 Flow cytometry analysis of CD11b expression in dHL-60 cells

To assess CD11b expression in dHL-60, cells were resuspended in pre-cooled phosphate-buffered saline (PBS) to achieve a single-cell suspension at a density of 1 × 10^6^ cells/ml. A 100 μL aliquot of this suspension was transferred to a flow cytometry tube for surface staining. To minimize nonspecific antibody binding to Fc receptors, 2 μl of CD16/32 (101,320, BioLegend, San Diego, USA) was added, and the cells were incubated at room temperature for 10 min. Following this, 1.25 μL of APC-conjugated anti-CD11b (101,212, BioLegend, San Diego, CA, USA) was added, and the mixture was incubated at 4°C for 30 min in the dark. After incubation, 1 ml of Flow Cytometry Staining Buffer (S1001, MULTI SCIENCES, Hangzhou, China) was added to gently resuspend the cells. The mixture was then centrifuged at 1200 rpm for 5 min, and the supernatant was discarded. The resulting cell pellet was resuspended in 200 μL of pre-cooled PBS and analyzed using a flow cytometer to quantify CD11b expression levels, providing insights into the activation status of neutrophil-like cells.

### 2.8 Validation of DEGs in dHL-60 cells and sample collection

To assess the differential expression of selected genes in dHL-60, the cells were allocated into two groups: the control group (dHL-60) and the treatment group, which received lipopolysaccharide (LPS) (L4391, Sigma-Aldrich, Lyon, France) at a concentration of 1 μg/ml. Both groups were seeded into 6-well plates at a density of 5 × 10⁵ cells/ml and incubated for 4 h at 37°C in a 5% CO₂ atmosphere. This setup allowed for the examination of transcriptional responses to LPS treatment in the context of neutrophil-like differentiation.

After incubation, the cell culture supernatants were carefully collected and stored at −80°C for subsequent ELISA analysis. The cells were harvested and centrifuged at 300 × g for 5 min. RNA extraction was carried out using TRIzol, followed by chloroform extraction, isopropanol precipitation, and an ethanol wash to ensure RNA purity. The concentration and purity of the extracted RNA were evaluated using NanoDrop spectrophotometer. Subsequently, 1 μg of RNA was reverse transcribed to generate complementary DNA (cDNA). Quantitative PCR was then performed using specific primers, following a rigorous protocol that included an initial denaturation step and 40 cycles of denaturation, annealing, and extension. The resulting data were analyzed to quantify relative gene expression levels, yielding valuable insights into the transcriptional alterations induced by LPS in neutrophil-like cells.

### 2.9 Enzyme-linked immunosorbent assay

Following the manufacturer’s protocols, ELISA kits (HM10120, HM11038, HM12170, HM11472, HM11143; Bioswamp, Wuhan, China) were employed to quantify the levels of LCN2, LTF, NE, CTSG, and CAMP in samples from MS patients, HC, and cell culture supernatants.

### 2.10 Statistical analysis

Statistical analyses were performed using SPSS 27.0 and GraphPad Prism 8.0 software. Data are expressed as mean ± standard deviation or as median with interquartile range. For normally distributed data, differences between groups were assessed using the t-test or Welch’s t-test; for non-normally distributed data, the Mann–Whitney U test was utilized. Prior to analysis, expression levels in the control group from the RT-qPCR results were normalized to 1. The receiver operating characteristic (ROC) curve was employed to evaluate the discriminative capacity of RNA between MS patients and healthy controls. Chi-square tests were applied for categorical factor analysis. A *p*-value of less than 0.05 was deemed statistically significant. Pearson rank correlation and Spearman correlation tests were used to assess the relationship between gene expression levels and Expanded Disability Status Scale (EDSS) scores.

## 3 Results

### 3.1 Demographic and clinical characteristics

In total, samples from 8 MS patients and 29 matched healthy controls were used in RNA sequencing. An additional cohort comprising 18 MS patients and 21 healthy controls was included for subsequent validation experiments. All participants were of Chinese Han ethnicity, originating from northern China. During the study visits, all patients in the validation cohort underwent comprehensive neurological examinations, which included assessment of disability severity using the EDSS and detailed evaluation of lesion distribution on T2-weighted magnetic resonance imaging. Detailed demographic and clinical profiles for the RNA-seq cohort are outlined in Table 1, while the characteristics of participants in the validation cohort are presented in Table 2.

**Table 1:** Demographic and clinical characteristics of the RNA-seq cohort.

	MS patients (*n* = 8)	HC (*n* = 29)	*p* value
Age, mean ± SD (years)	41.88 ± 18.39	49.34 ± 11.91	0.306
Female, *n* (%)	6 (75.00%)	22 (75.86%)	0.960
Age at disease onset, mean ± SD (in year)	39.13 ± 16.90	NA	NA
Clinical symptom at onset			
Optic neuritis, *n* (%)	1 (12.50%)	NA	NA
Acute myelitis, *n* (%)	5 (62.50%)	NA	NA
Brain attacks, *n* (%)	0 (0.00%)	NA	NA
Mix attacks, *n* (%)	2 (25.00%)	NA	NA
WBC count (×10^9^) median, (IQR)	7.99 (12.05–5.92)	5.31 (6.28–4.71)	0.039
Neutrophil count (×10^9^) median, (IQR)	4.10 (7.91–3.40)	2.97 (3.83–2.38)	0.027
Lymphocyte counts (×10^9^) median, (IQR)	1.49 (1.85–1.16)	1.77 (2.03–1.58)	0.061
NLR median, (IQR)	2.70 (7.52–1.93)	1.59 (2.37–1.38)	0.024
NWR median, (IQR)	0.65 (0.80–0.58)	0.55 (0.63–0.52)	0.039

MS, multiple sclerosis; HC, healthy control; WBC, white blood cell; NLR, Neutrophil to lymphocyte ratio; NWR, Neutrophil to white blood cell ratio; Brain attacks, including brainstem and brain attacks; NA, not applicable; SD, standard deviation; IQR, Interquartile range.

**Table 2:** Demographic and clinical characteristics of the validation cohort.

	MS patients (*n* = 18)	Control (*n* = 21)	*p* value
Age, mean ± SD (years)	37.22 ± 11.13	42.19 ± 13.41	0.221
Female, *n* (%)	12 (66.67%)	16 (76.19%)	0.510
Age at disease onset, mean ± SD (in year)	34.72 ± 10.16	NA	NA
Clinical symptom at onset			
Optic neuritis, *n* (%)	1 (5.56%)	NA	NA
Acute myelitis, *n* (%)	10 (55.56%)	NA	NA
Brain attacks, *n* (%)	2 (11.11%)	NA	NA
Mix attacks, *n* (%)	5 (27.78%)	NA	NA
EDSS score, mean ± SD	3.06 ± 1.62	NA	NA
Location of lesions on T_2_-weighted images			
Periventricular	9 (50%)	NA	NA
Juxtacortical	9 (50%)	NA	NA
Infratentorial	10 (55.56%)	NA	NA
Spinal cord (cervical)	15 (83.33%)	NA	NA
Spinal cord (thoracic)	12 (66.67%)	NA	NA
WBC count (×10^9^) median, (IQR)	7.25 (11.45–5.43)	5.46 (6.45–4.80)	0.007
Neutrophil count (×10^9^) median, (IQR)	5.29 (9.03–3.51)	3.14 (3.78–2.81)	0.006
Lymphocyte counts (×10^9^) median, (IQR)	1.50 (1.94–1.08)	1.70 (2.06–1.40)	0.135
NLR median, (IQR)	3.40 (6.96–2.15)	1.71 (2.62–1.36)	0.003
NWR median, (IQR)	0.71 (0.83–0.63)	0.56 (0.66–0.51)	0.009

MS, multiple sclerosis; HC, healthy control; EDSS, expanded disability status scale; WBC, white blood cell; NLR, Neutrophil to lymphocyte ratio; NWR, Neutrophil to white blood cell ratio; Brain attacks, including brainstem and brain attacks; NA, not applicable; SD, standard deviation; IQR, Interquartile range.

### 3.2 Transcriptomic analysis of peripheral blood neutrophils during the onset of MS

**Figure 1:**
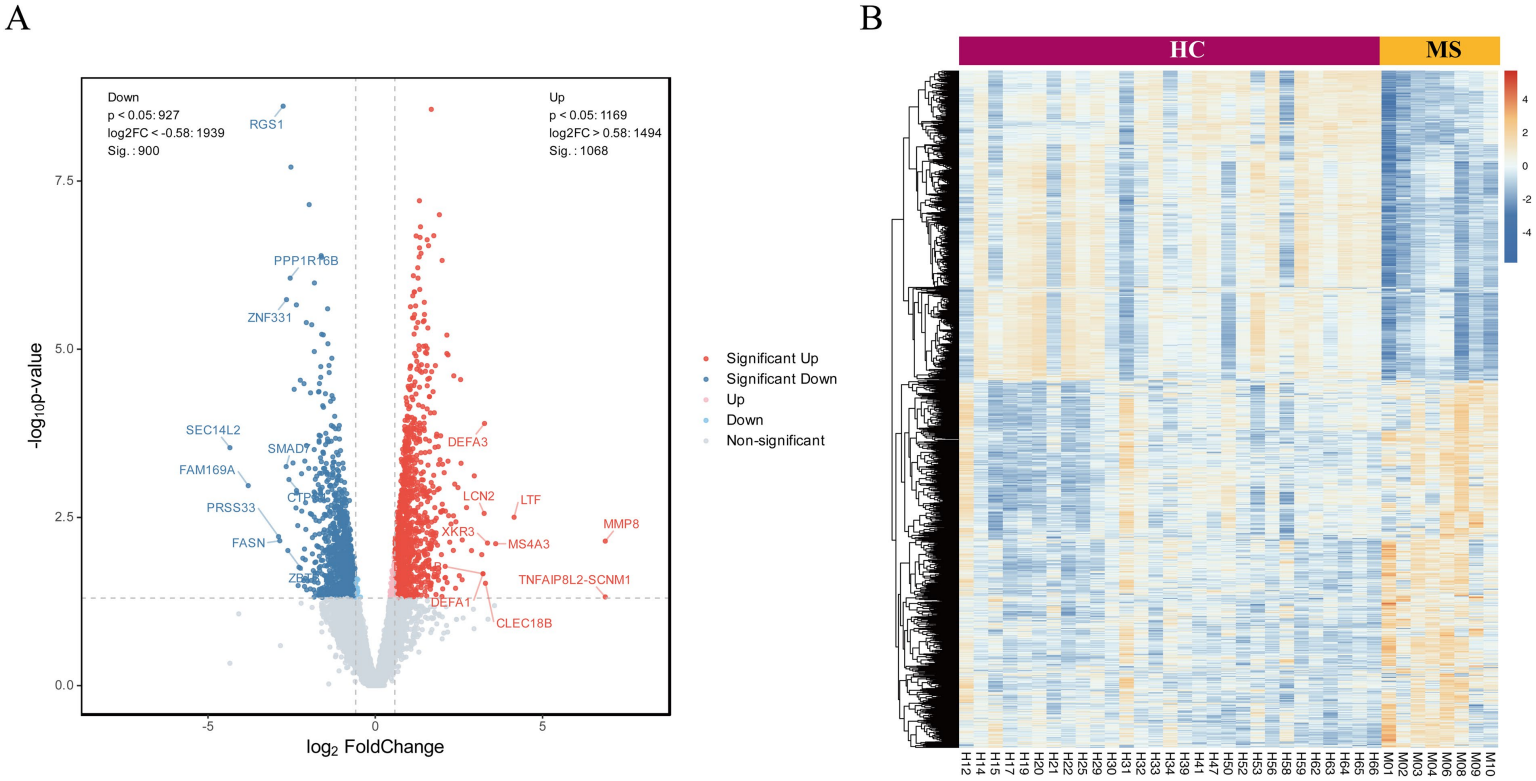
Analysis of DEGs in neutrophils from peripheral blood of MS patients and healthy controls. **(A)** The volcano plot illustrates the expression profiles of differentially expressed RNAs, with red points denoting upregulated RNAs, blue points representing downregulated RNAs, and gray points indicating RNAs exhibiting no statistically significant differences. **(B)** Unsupervised hierarchical clustering groups of DE RNAs between groups.

Transcriptomic sequencing of peripheral blood neutrophils from MS patients revealed significant alterations in mRNA expression compared to HC, identifying a total of 1,968 differentially expressed genes (*p* < 0.05; |Fold Change| > 1.5). Among these, 1,068 genes were upregulated, including *DEFA3*, *LCN2*, *LTF*, and *MMP8*, which are associated with neutrophil activation and inflammatory processes. In contrast, 900 genes were downregulated, such as *RGS1*, *PPP1R6B*, and *ZNF331*, indicating the suppression of pathways potentially relevant to MS pathophysiology. Genes with the greatest statistical significance and largest expression changes are positioned at the extremes of the plot, underscoring their potential as key regulators of disease processes. The central cluster of points represents genes with minimal expression changes, highlighting the specificity of the observed transcriptomic alterations (Figure 1A).To visualize the comprehensive changes in gene expression, hierarchical clustering was employed to generate volcano plots and heatmaps, effectively illustrating the differential transcriptomic landscape associated with MS onset (Figure 1B).

### 3.3 Functional enrichment analysis of DEGs

**Figure 2:**
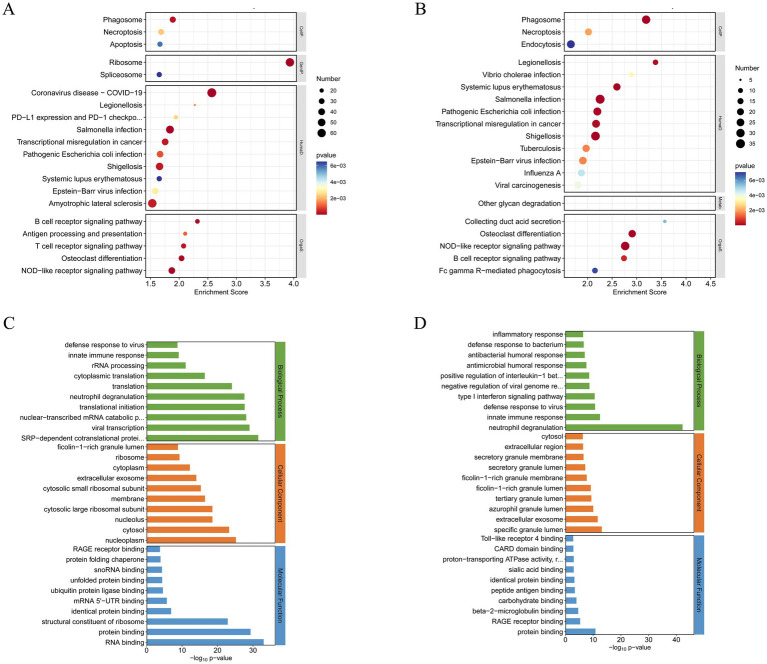
GO and KEGG functional enrichment analysis of differentially expressed mRNAs (|Fold Change| > 1.5). **(A)** Top 20 KEGG pathway of total DEGs (ranked by their enrichment *p* value). **(B)** Top 20 KEGG pathway of upregulated DEGs (ranked by their enrichment *p* value). **(C)** Top 30 GO terms of total DEGs. **(D)** Top 30 GO terms of upregulated DEGs.

We conducted KEGG functional enrichment analysis on the DEGs, focusing on both the overall dataset and the subset of upregulated genes (Figures 2A,B). The bubble plots highlight the top 30 enriched pathways and associated diseases, with prominent representation of immune-related signaling pathways such as phagosome formation, necroptosis, and antigen processing and presentation. Key adaptive and innate immune pathways, including the B cell receptor signaling pathway, T cell receptor signaling pathway, and NOD-like receptor signaling pathway, were also significantly enriched. These findings implicate a complex interplay of immune mechanisms in the pathogenesis of MS. Furthermore, diseases characterized by immune dysregulation, such as systemic lupus erythematosus and Epstein–Barr virus infection, were notably enriched, underscoring the relevance of systemic immune perturbations in MS.

To further investigate functional alterations, GO analysis was conducted to examine the biological processes, cellular components, and molecular functions associated with DEGs (Figure 2C). Biological processes were predominantly enriched for immune responses, intracellular signal transduction, and neutrophil degranulation. Cellular component analysis revealed significant enrichment in cytosolic and extracellular regions, particularly components involved in protein transport and secretion. Molecular functions included RNA binding and structural constituents of ribosomes, suggesting widespread changes in protein synthesis and immune-related transcriptional activity.

GO analysis of the upregulated genes offered additional resolution (Figure 2D), revealing strong enrichment in biological processes associated with inflammatory responses, type I interferon signaling pathways, and neutrophil degranulation. Among these, neutrophil degranulation demonstrated the most significant enrichment, highlighting its central role in the innate immune response. This process, which involves the release of antimicrobial and pro-inflammatory mediators, is a key effector function of neutrophils. Its robust enrichment underscores its pivotal contribution to MS pathogenesis, potentially amplifying inflammatory cascades and driving tissue damage within the CNS. These findings illuminate critical molecular pathways and immune mechanisms underlying disease progression, providing a foundation for future mechanistic investigations.

**Figure 3:**
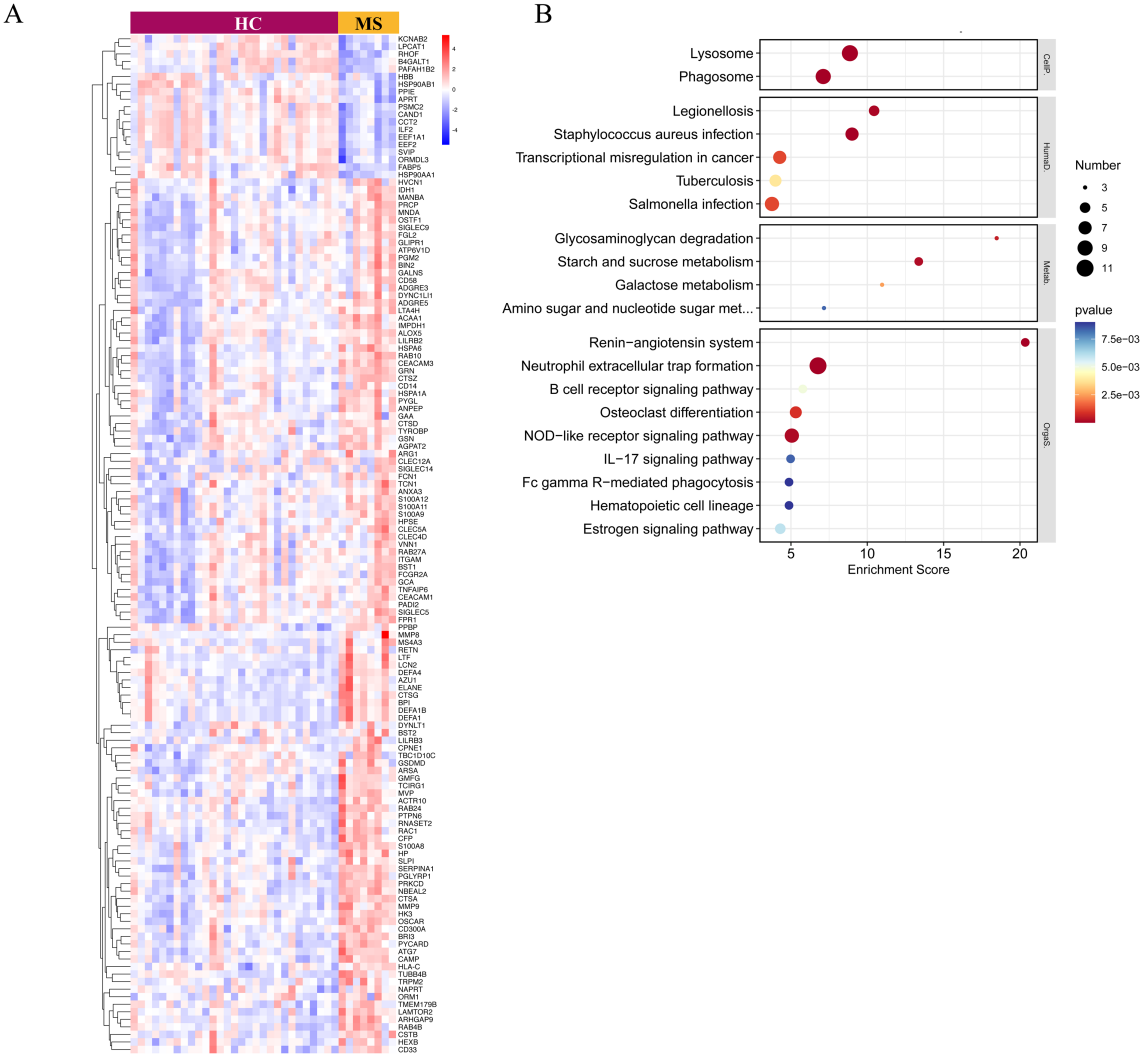
Gene expression and KEGG functional enrichment analysis of upregulated genes associated with neutrophil degranulation. **(A)** The heatmap illustrates the expression levels of genes linked to neutrophil degranulation, providing a visual representation of their differential expression across samples. **(B)** The KEGG pathway analysis highlights the pathways significantly associated with the upregulated genes involved in neutrophil degranulation.

To further elucidate the involvement of neutrophil degranulation, we performed a heatmap analysis of relevant genes (Figure 3A). Additionally, KEGG functional enrichment analysis of these upregulated genes revealed associations with critical biological processes, including the formation of NETs, NOD-like receptor signaling pathways, and the IL-17 signaling pathway (Figure 3B).

**Figure 4:**
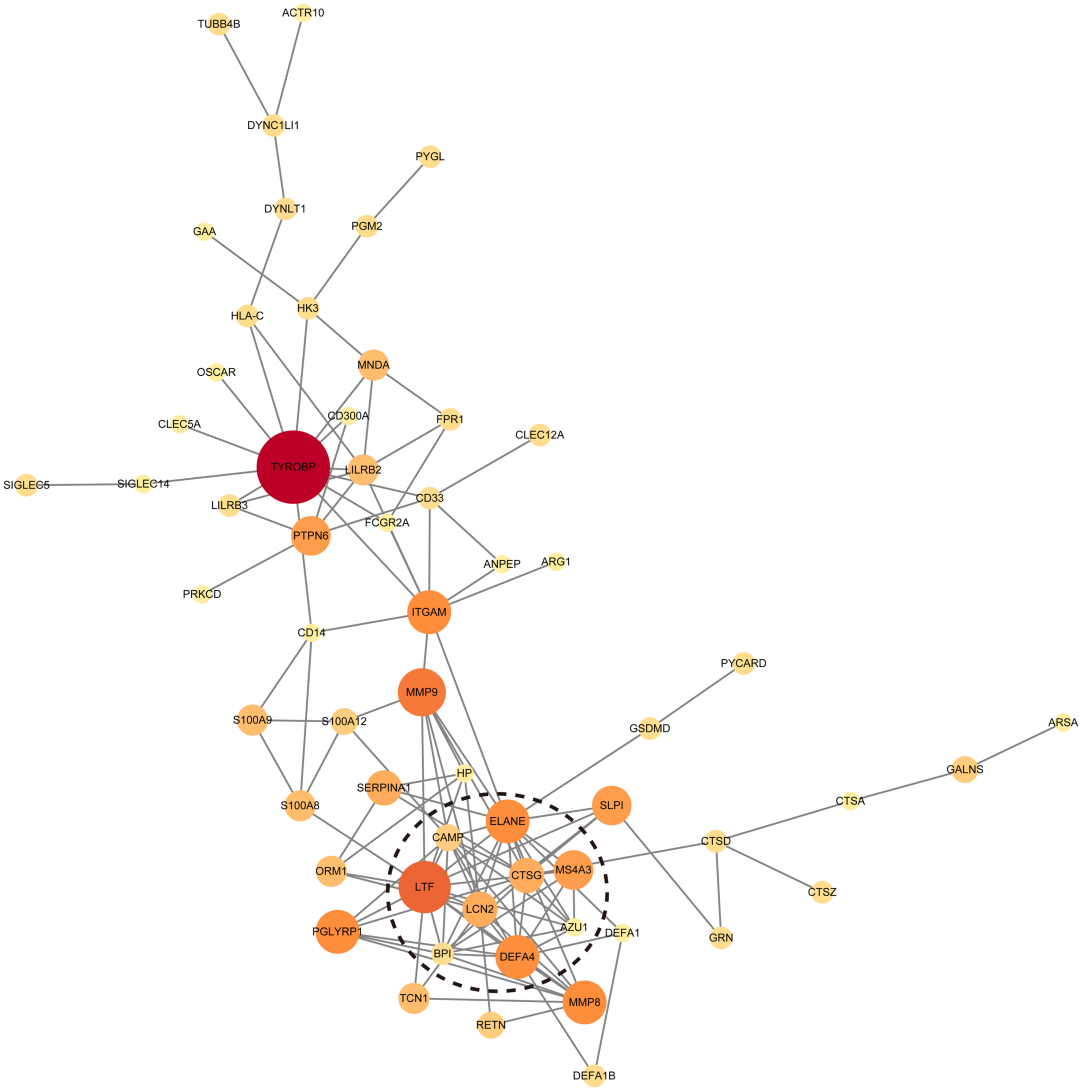
Protein–Protein Interaction (PPI) network of upregulated genes associated with neutrophil degranulation. The dotted circle highlights the cluster with the highest MCODE score (≥5), indicating a tightly interconnected group of genes that may play a crucial role in the underlying biological processes.

To visualize the interactions among these genes, we utilized Cytoscape software to construct a protein–protein interaction (PPI) network for the upregulated genes associated with neutrophil degranulation (Figure 4). This analysis identified two highly interconnected subnetworks through the MCODE plugin, with one network exhibiting an MCODE score exceeding 5. This network comprises key genes, including *LTF*, *LCN2*, *ELANE*, *CAMP*, *CTSG*, *MS4A3*, *DEFA4*, *AZU1*, and *BPI*, highlighting their potential collaborative roles in the inflammatory milieu characteristic of MS.

### 3.4 RT-qPCR validation and clinical relevance of DEGs

To validate our findings, five genes—*LCN2*, *LTF*, *ELANE*, *CAMP*, and *CTSG*—were selected based on the criteria outlined in Section 2.3 for subsequent RT-qPCR analysis. The specific primers utilized in this study are shown in Table 3.

**Table 3:** PCR primers used for amplification.

Gene	Forward	Reverse
GAPDH	GGAAGCTTGTCATCAATGGAAATC	TGATGACCCTTTTGGCTCCC
LCN2	GACAACCAATTCCAGGGGAAG	CAGGACGGAGGTGACATTGTAG
LTF	GGAAAGGACAAGTCACCGAAAT	AGTTCTGGATGGCAGTGAAGTAGC
ELANE	TCGCAGCAACGTCTGCACT	TCGGAGCGTTGGATGATAGA
CAMP	GGGGCTCCTTTGACATCAGT	GGTAGGGCACACACTAGGAC
CTSG	TCCAGAGACGGGAAAACACC	CCGTCTGACTCTTCTGCTCA

**Figure 5:**
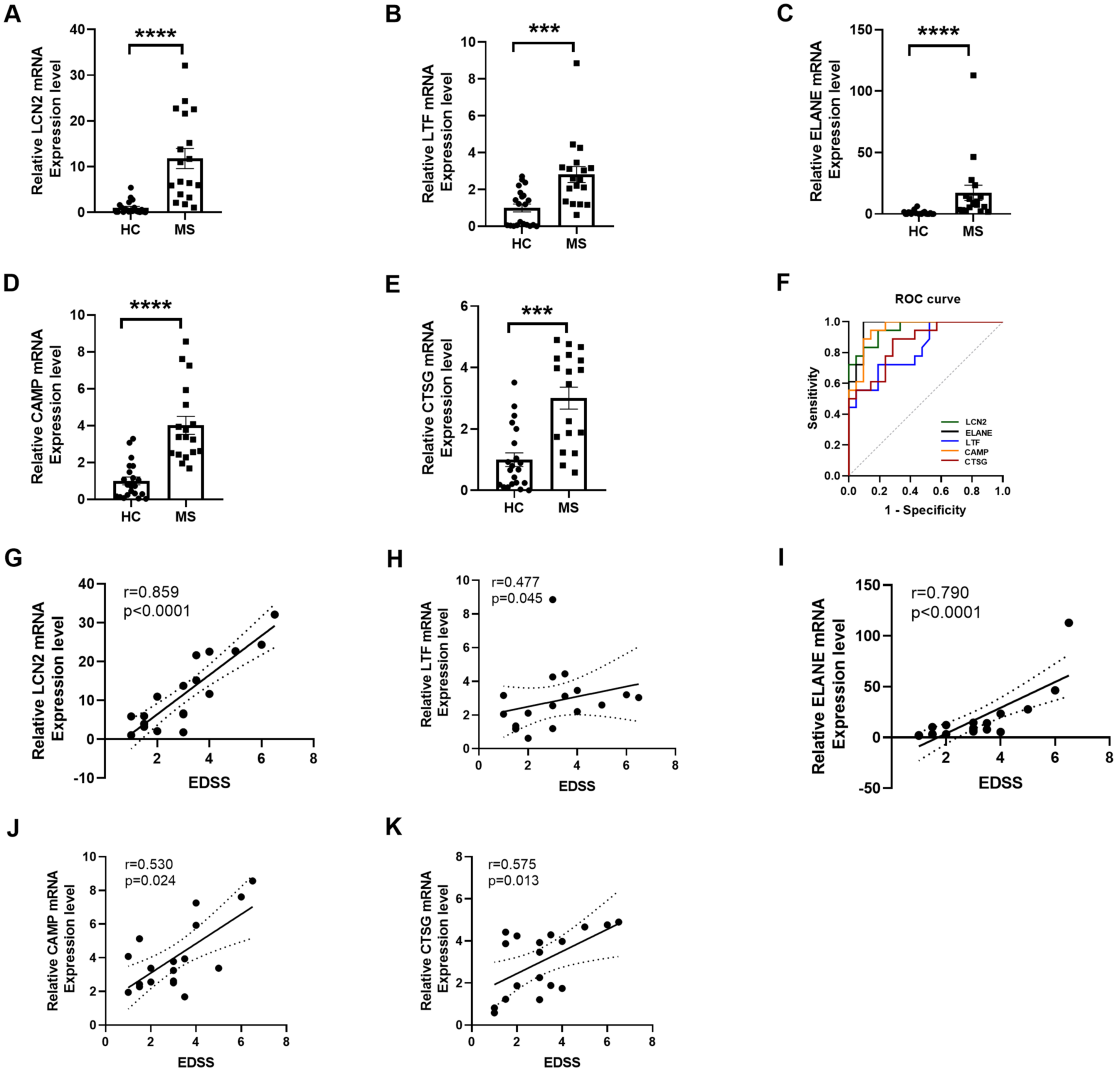
RT-qPCR validation and ROC curve of selected DE RNAs. **(A–E)** RT-qPCR validation of five selected differentially expressed mRNAs demonstrates significant alterations in expression levels. **(F)** ROC curve analysis evaluates the diagnostic efficacy of these five significant DE mRNAs, indicating their potential as biomarkers for distinguishing MS from HC. **(G–K)** The correlation analysis illustrates the relationship between mRNA expression levels of *LCN2*, *LTF*, *ELANE*, *CAMP*, and *CTSG* genes and EDSS scores. Statistical significance is denoted by *** for *p* < 0.001 and **** for *p* < 0.0001.

This validation revealed significant differences in the RNA expression levels of *LCN2*, *LTF*, *ELANE*, *CAMP*, and *CTSG* between MS patients and HCs (Figures 5A–E). Further analysis using ROC curves demonstrated the diagnostic potential of these genes in distinguishing MS from HC. Specifically, *LCN2* exhibited an area under the curve (AUC) ranging from 0.8940 to 1.0000 (*p* < 0.0001), while *LTF* showed an AUC of 0.6990 to 0.9540 (*p* = 0.0005). *ELANE* presented an AUC between 0.9200 and 1.0000 (*p* < 0.0001), *CAMP* ranged from 0.8870 to 1.0000 (*p* < 0.0001), and *CTSG* had an AUC of 0.7510 to 0.9740 (*p* = 0.0001) (Figure 5F, Table 4). These results underscore the potential of *LCN2*, *LTF*, *ELANE*, *CAMP*, and *CTSG* as promising biomarkers for the diagnosis of MS.

**Table 4:** ROC curve of selected DE RNAs.

Gene	AUC	95%CI	*p* value	Cut off value	Youden Index
LCN2	0.952	0.894–1.000	<0.0001	3.6098	0.841
LTF	0.827	0.699–0.954	0.0005	1.9435	0.532
ELANE	0.968	0.920–1.000	<0.0001	1.7820	0.905
CAMP	0.950	0.887–1.000	<0.0001	1.8811	0.801
CTSG	0.862	0.751–0.974	0.0001	1.1258	0.603

We further investigated the relationship between gene expression levels and the EDSS scores in patients with MS. Notably, *LCN2* expression exhibited a robust positive correlation with EDSS scores (r = 0.859, *p* < 0.0001), indicating a strong association with disease severity (Figures 5G–K). Similarly, *ELANE* expression was significantly correlated with EDSS (r = 0.790, *p* < 0.0001), while moderate correlations were observed for *LTF* (r = 0.477, *p* = 0.045), *CAMP* (r = 0.530, *p* = 0.024), and *CTSG* (r = 0.575, *p* = 0.013). These findings highlight the potential utility of *LCN2*, *ELANE*, *CAMP*, and *CTSG* as biomarkers for assessing neurological disability in MS, with *LTF* providing additional, albeit more moderate, predictive value. Collectively, these data reinforce the critical role of these genes in reflecting disease progression and severity in MS.

### 3.5 Serum protein alterations of LCN2, LTF, ELANE, CAMP, and CTSG in MS

**Figure 6:**
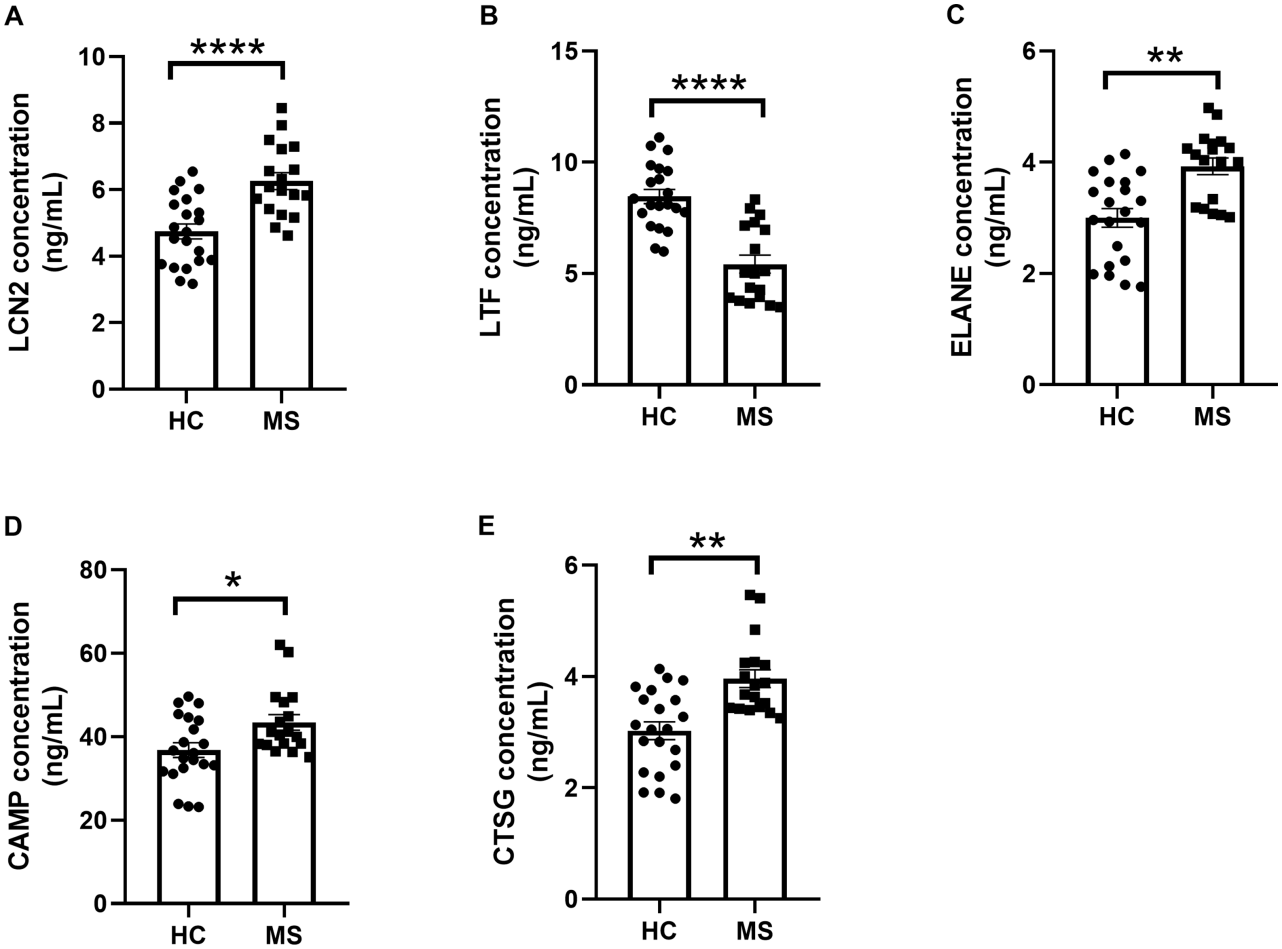
ELISA analysis of serum LCN2, LTF, ELANE, CAMP, and CTSG protein levels. **(A–E)** ELISA-based analysis of protein level alterations in LCN2, LTF, ELANE, CAMP, and CTSG in the serum of MS patients and HC. * represents *p* < 0.05, ** represents *p* < 0.01, **** represents *p* < 0.001.

ELISA quantification revealed significant differences in serum protein levels of LCN2, LTF, ELANE, CAMP, and CTSG between MS patients and HC. Serum levels of LCN2 (p < 0.0001), ELANE (*p* < 0.01), CAMP (*p* < 0.05), and CTSG (p < 0.01) were elevated in MS patients, while LTF levels were significantly reduced (p < 0.0001) (Figures 6A–E). The discrepancy between LTF gene expression and serum protein levels suggests post-transcriptional modifications influencing LTF regulation in MS.

### 3.6 Validation of DEGs in HL-60 cell lines

**Figure 7:**
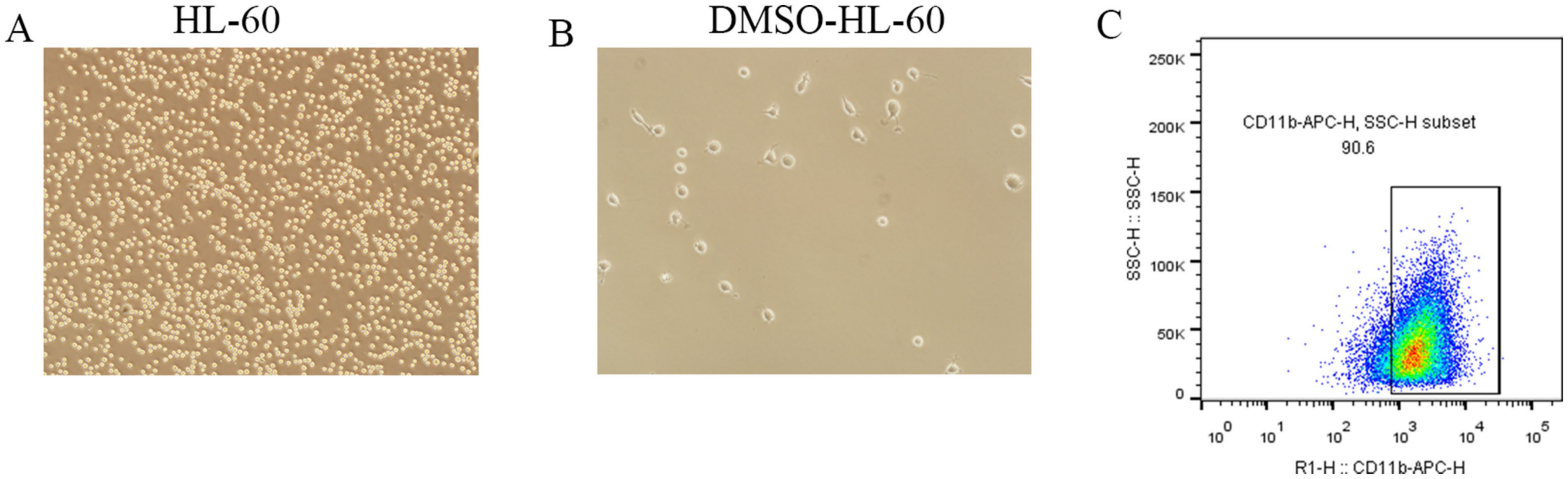
Morphological changes and surface marker expression in dHL-60 cells. **(A,B)** Observation of morphological changes in DMSO-induced HL-60 cells differentiating into dHL-60 cells using a conventional optical microscope. **(C)** Flow cytometry analysis of CD11b expression in dHL-60 cells.

To further elucidate the expression of the selected DEGs in neutrophils, we employed the HL-60 cell line for *in vitro* studies. Following a three-day induction with 1.25% DMSO, morphological changes in the HL-60 cells were assessed using standard optical microscopy. The differentiated HL-60 cells (dHL-60) exhibited notable changes, including increased size, enhanced adhesion, and greater morphological diversity compared to the untreated HL-60 cells (Figures 7A,B).

**Figure 8:**
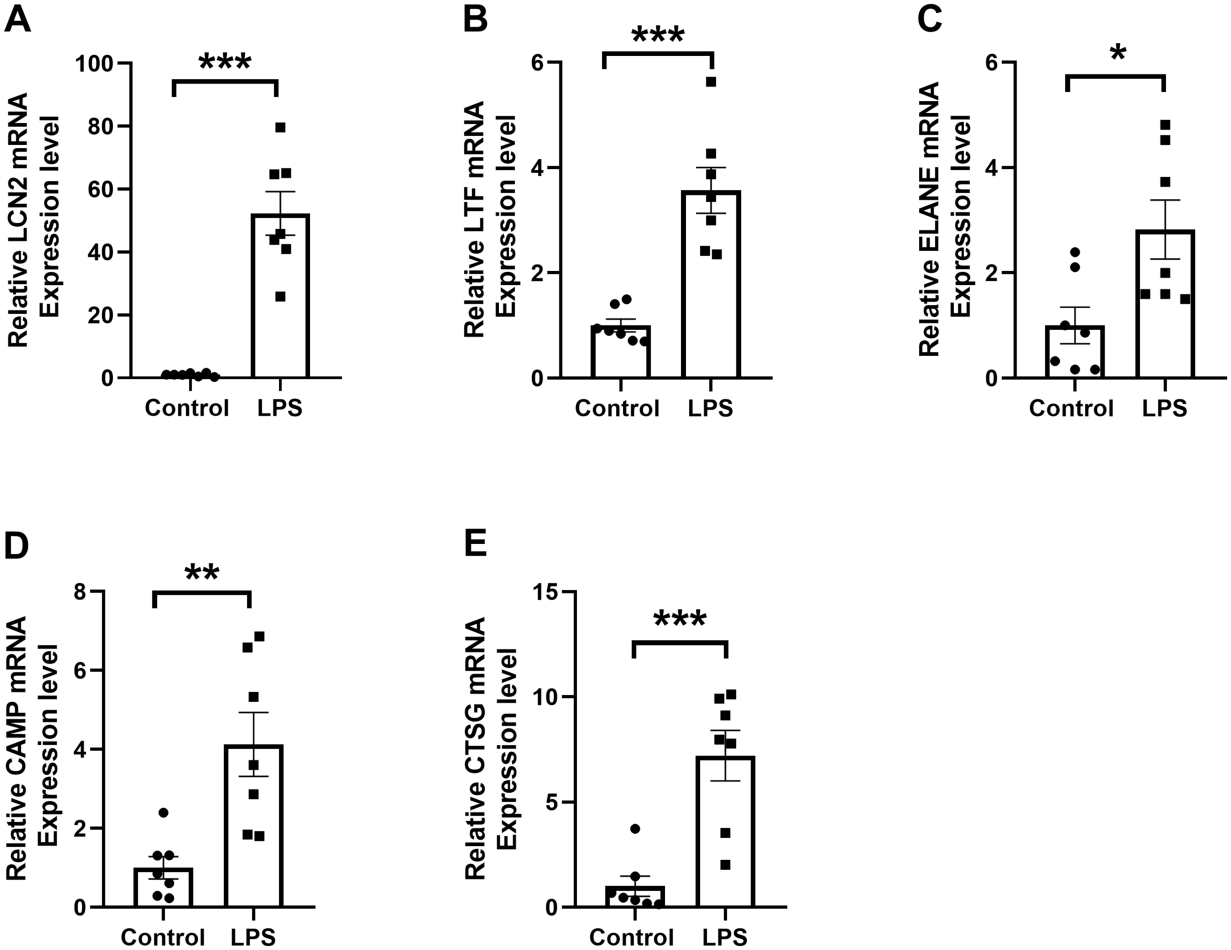
RT-qPCR validation of differential RNA expression in dHL-60 cells. **(A–E)** Reletive mRNA levels of the five selected DE mRNAs. * represents *p* < 0.05, ** represents *p* < 0.01, *** represents *p* < 0.001.

Flow cytometry analysis confirmed that over 90% of the dHL-60 cells expressed CD11b on their surface (Figure 7C), validating their suitability for subsequent experiments. RT-qPCR results revealed a significant upregulation of *LCN2*, *LTF*, *ELANE*, *CAMP*, and *CTSG* in the LPS-treated (1 μg/ml) dHL-60 cells compared to the untreated dHL-60 group (Figures 8A–E). These findings reinforce the relevance of these genes in the context of neutrophil activation and response.

### 3.7 ELISA analysis of protein expression in cell culture supernatants

The protein levels of LCN2, LTF, ELANE, CAMP, and CTSG in cell culture supernatants were quantified using ELISA. The results demonstrated that LCN2 (*p* < 0.05), ELANE (*p* < 0.01), CAMP (*p* < 0.01), and CTSG (*p* < 0.05) were significantly elevated in the supernatants of LPS-treated (1 μg/ml) dHL-60 cells compared to untreated dHL-60 cells (Fig. S1A), whereas LTF (*p* < 0.05) protein expression was significantly decreased in the LPS-treated group compared to the untreated group (Fig. S1B).

## 4 Discussion

Our study employed RNA sequencing to profile the mRNA expression of peripheral blood neutrophils in MS patients, revealing significant transcriptional alterations. A total of 1,968 differentially expressed mRNAs (*p* < 0.05, |Fold Change| > 1.5) were identified, with 1,068 upregulated and 900 downregulated. We prioritized the analysis of upregulated genes because they are closely associated with the activated state of neutrophils and the exacerbation of inflammation, as well as being enriched in pathways critical to MS pathogenesis, such as degranulation, NETs formation, and immune signaling. These extensive transcriptional changes underscore the pivotal role of neutrophils in MS pathogenesis and provide novel insights into their contribution to disease mechanisms.

### 4.1 Neutrophils as key mediators in MS pathogenesis

GO and KEGG enrichment analyses demonstrated that DEGs were predominantly associated with processes such as phagocytosis and anti-infective responses, including pathways related to antigen processing and presentation, B cell receptor signaling, T cell receptor signaling, and NOD-like receptor pathways. The significant enrichment of upregulated genes in neutrophil degranulation underscores its potential importance in the inflammatory milieu of MS.

Recent literature has increasingly highlighted the involvement of neutrophils in autoimmune diseases, including MS (23). The activation phenotype of neutrophils is markedly enhanced in the peripheral blood of patients with MS (24). Circulating levels of neutrophil-activating chemokines and neutrophil-derived enzymes—including CXCL1, CXCL8, NE, and MPO—are significantly elevated in MS and correlate with the formation of new inflammatory lesions (25–27). The NLR, a potential marker of disease activity, is consistently higher in MS patients, with a pronounced increase observed during relapses compared to remission (14, 28). Moreover, neutrophils isolated from relapsing–remitting MS patients exhibit heightened expression of pro-inflammatory markers and an enhanced resistance to apoptosis, underscoring their contribution to disease pathophysiology (24, 29).

Neutrophils have also been identified in the CSF of MS patients during early disease stages and at the onset of relapses, highlighting their active involvement in disease initiation (30). Intriguingly, this phenomenon diminishes with disease progression, suggesting a more prominent role for neutrophils in the initial and relapsing phases of MS. On a genetic level, *Neutrophil Cytosolic Factor 4* (*NCF4*), which encodes a subunit of the nicotinamide adenine dinucleotide phosphate (NADPH) oxidase complex in neutrophils, has emerged as a genetic susceptibility locus for MS in a recent genome-wide association study by the International Multiple Sclerosis Genetics Consortium (31). These findings collectively underscore the multifaceted role of neutrophils in driving MS pathogenesis and highlight their potential as therapeutic targets.

Our analysis indicated an increase in both neutrophil proportions and NLR in patients during the acute phase of MS, aligning with a state of heightened activation. The enrichment of DEGs involved in neutrophil degranulation suggests that these cells may release proteolytic enzymes (e.g., NE, MPO) and reactive oxygen species (ROS), contributing to tissue damage and exacerbated inflammation (32, 33). Such processes may disrupt immune homeostasis, leading to neurological damage and the amplification of disease severity.

### 4.2 Immune interactions and antigen presentation

MS is recognized as a CNS autoimmune disorder involving a complex interplay among various immune cell types, notably T and B cells (34). Activated CD4+ T cells, particularly Th1 and Th17 subsets, play a major role in driving inflammatory responses through cytokines such as IFN-*γ* and IL-17, which contribute to myelin damage and promote the activation of other immune cells (35). B cells, through their functions as antigen-presenting cells (APCs) and producers of autoantibodies, further amplify the autoimmune cascade (36).

Our functional analysis of RNA revealed significant enrichment of genes linked to antigen processing and presentation pathways, as well as T and B cell receptor signaling. Notably, neutrophils, upon migration to lymph nodes, can express CXCR4, MHC II, and co-stimulatory molecules, enabling them to present antigens to CD4+ T cells and facilitate their differentiation into Th17 cells (37). This interaction underscores the capacity of neutrophils to act as modulators of adaptive immunity in autoimmune settings. Th17 cells, characterized by their secretion of IL-17, are pivotal for neutrophil recruitment (38), creating a potential feedback loop that amplifies inflammation.

Further, during inflammatory responses, neutrophils release cytokines such as BAFF (B cell activating factor) and APRIL, which activate B cells and enhance immune responses against myelin antigens (39, 40). This intricate network of immune interactions highlights the multifaceted role of neutrophils in the pathogenesis of MS.

### 4.3 NETs and MS

In recent years, NETs has been found to be associated with a variety of autoimmune diseases, such as systemic lupus erythematosus (41), rheumatoid arthritis, cystic fibrosis (42, 43) and psoriasis (44). Research specifically examining NETs in MS is limited, though elevated levels of MPO-DNA complexes, a marker for NETs, have been detected in the serum of MS patients (45). While these levels do not consistently correlate with disease activity, there is evidence of gender-specific differences, with higher levels in male patients (46). This suggests a potential role for NETs in sex-based variations in MS pathogenesis.

### 4.4 Implications of NETs in MS pathophysiology

Our GO functional enrichment analysis of upregulated genes revealed significant associations with processes involved in NETs formation. While the connection between NETs and autoimmunity is well-documented, the specific mechanisms underlying their role in MS remain elusive. IL-17-producing Th17 cells, critical in many autoimmune diseases (47), have been shown to interact with NETs. Wilson et al. demonstrated that NETs can directly stimulate T cell activation by mediating STAT3 phosphorylation through histone-TLR2 interactions on CD4+ T cells, promoting their differentiation into Th17 cells (48). This connection suggests that NETs could potentially exacerbate autoimmune responses in MS by fostering T cell-driven inflammation.

Given the ability of NETs to serve as both initiators and amplifiers of immune responses (84), future research aimed at understanding their contribution to MS pathophysiology could uncover new therapeutic targets. In animal models such as EAE, investigations into the pathogenic roles of NETs could provide insights into how they might be manipulated to mitigate disease progression.

### 4.5 Potential biomarkers and therapeutic targets

Among the differentially expressed genes, *LCN2*, *LTF*, *ELANE*, *CAMP*, and *CTSG* were identified as central nodes in the PPI network, suggesting their relevance as biomarkers and therapeutic targets in immune and inflammatory responses.

The *LCN2* gene, also known as lipocalin 2 or neutrophil gelatinase-associated lipocalin (NGAL), has emerged as a significant biomarker for a range of conditions, including acute kidney injury (49), Alzheimer’s disease (50), MS (51) and depression (52). LCN2 may exert both protective and detrimental effects on neural cells by modulating inflammatory responses and iron metabolism, with the specific mechanisms likely varying according to the disease stage or pathological context (53). Research in EAE has demonstrated that LCN2 influences disease severity, T cell proliferation, and demyelination (51, 53–55). Notably, elevated levels of LCN2 have been observed in the blood and cerebrospinal fluid of MS patients. Consistent with these findings, our study revealed significantly higher *LCN2* mRNA levels in peripheral blood neutrophils and elevated serum LCN2 protein levels in MS patients compared to healthy controls. *Lcn2*-knockout mice demonstrate attenuated EAE severity, characterized by reduced inflammatory cell infiltration, demyelination, and cytokine production, as well as impaired T-cell activation and Th17/Th1 polarization (53, 55, 56). Neutrophils, the primary source of LCN2, facilitate adaptive immunity by activating antigen-presenting cells and promoting T-cell differentiation (57–59). Notably, LCN2 interacts with dendritic cells via 24p3R, influencing antigen presentation during EAE progression (60). Pharmacological interventions such as natalizumab reduce LCN2 expression and mitigate EAE severity, underscoring its pro-inflammatory role (51).

In progressive MS, LCN2 plays a critical role in regulating CNS inflammation, inducing neuronal cell death, modulating dendritic spine formation, and controlling iron deposition and cellular transfer, thereby participating in both demyelination and neurodegeneration. Studies indicate a correlation between LCN2 levels and MS severity (54, 60–65), underscoring its potential as a biomarker and therapeutic target. Future research should further elucidate its specific mechanisms in the pathogenesis of MS and explore LCN2-based therapeutic strategies.

The *ELANE* gene encodes NE, a serine protease secreted by neutrophils that plays a vital role in host defense and inflammatory responses. NE is indispensable for the formation of NETs. Notably, inhibition of NE has been shown to attenuate neutrophil infiltration into the optic nerve during EAE. This finding underscores the relevance of NE to the pathological mechanisms underlying MS (66). In the present study, we observed significantly elevated *ELANE* expression in neutrophils, as determined by RT-qPCR, and elevated ELANE protein levels in serum, as measured by ELISA. Furthermore, the expression of *ELANE* in neutrophils was positively correlated with the EDSS. These findings align with previous studies reporting that the expression of CXCL1, CXCL5, and NE is closely associated with the clinical and radiological features of CNS damage in MS. Additionally, the expression of CCL2, CXCL1, CXCL5, and NE correlates with EDSS scores (17), further supporting the pivotal role of *ELANE* in MS pathogenesis.Therapeutically, sivelestat—a selective NE inhibitor approved for the treatment of acute respiratory distress syndrome—has demonstrated potential in alleviating neuropathic pain by limiting T-cell infiltration (67). Chronic neuropathic pain, a debilitating symptom for many MS patients, highlights the rationale for repurposing sivelestat as a candidate therapy for MS (68). Mechanistically, selective NE inhibition disrupts neutrophil migration, impairs phagocytic activity, and prevents the formation of NETs (69–71). Of note, emerging evidence indicates that sivelestat also exhibits therapeutic promise in acute neuromyelitis optica spectrum disorders (NMOSD) and other neuroinflammatory diseases, further broadening its clinical applicability (72). Targeting *ELANE* represents a promising therapeutic avenue, offering novel opportunities for personalised interventions in MS and other neuroinflammatory disorders.

LTF is a multifunctional glycoprotein known for its antifungal, antimicrobial, and anticancer properties (73). In this study, we observed reduced LTF protein expression in the serum of MS patients, corroborating previous reports. LTF has been explored as a therapeutic agent for neurodegenerative diseases, including Alzheimer’s disease, Parkinson’s disease, and MS (74, 75). Notably, early investigations have demonstrated that oral administration of LTF significantly mitigates MS disease progression, suppresses inflammatory cytokines, and reduces CNS damage (75). Beyond these effects, LTF’s role as an iron-binding protein is particularly relevant in MS, where it chelates and sequesters excess iron released due to oligodendrocyte damage (76). Interestingly, our study revealed a discrepancy between *LTF* mRNA expression in neutrophils and its protein levels in serum and ex vivo culture supernatants, suggesting the involvement of complex regulatory mechanisms. These may include mRNA-specific degradation, translational repression, or post-translational modifications leading to protein degradation.

The *CAMP* gene encodes the antimicrobial peptide cathelicidin, with LL-37 being its active form in humans (77). Research indicates that LL-37 can drive the development of EAE in mice by influencing Th17 differentiation (78, 79). Moreover, LL-37 plays multiple roles in NETs formation, enhancing antimicrobial activity while modulating immune responses and inflammatory processes critical to MS (80). The results of the present study further showed that the gene expression level of *CAMP* in neutrophils from MS patients was positively correlated with EDSS scores, suggesting its potential role in disease severity. With further research, targeting *CAMP* to regulate LL-37 function may provide a new therapeutic strategy for MS.

CTSG functions as a key protease in the degradation of myelin basic protein, potentially disrupting immunodominant myelin epitopes and allowing autoreactive T cells to evade negative selection. This mechanism may contribute to the pathogenesis of MS (81, 82). In the present study, we observed significantly elevated *CTSG* mRNA expression in peripheral blood neutrophils and increased serum protein levels of CTSG in MS patients compared to HC. Research on CTSG in the context of MS is still nascent, necessitating further exploration of its impact on inflammation, blood–brain barrier integrity, and immune responses.

Collectively, our findings identify *LCN2*, *ELANE*, *CAMP*, and *CTSG* as compelling biomarkers for the evaluation of neurological disability in MS, with *LTF* offering complementary, though comparatively moderate, predictive value. The robust associations between these gene expression levels and EDSS scores underscore their potential clinical relevance in monitoring disease progression. Further investigations are needed to delineate their mechanistic roles in MS pathogenesis and to assess their feasibility as therapeutic targets.

### 4.6 Limitations, implications, and future directions

This study has certain limitations. The relatively small sample size may impact the statistical significance and representativeness of our results, underscoring the need for further investigation in larger cohorts. Additionally, while our focus was on MS, investigating related autoimmune conditions such as neuromyelitis optica spectrum disorder (NMOSD) and myelin oligodendrocyte glycoprotein antibody disease (MOGAD) could offer comparative insights and deepen our understanding of neutrophil involvement across similar diseases. Furthermore, the observed upregulation of genes in MS may reflect both immune activation and disease-specific mechanisms. Further studies comparing other inflammatory diseases and conducting functional experiments are necessary to clarify their MS-specific roles. The influence of environmental and genetic factors on gene expression was not fully explored, highlighting an area for future research to better contextualize the observed transcriptional changes.

Despite these limitations, this study makes a significant contribution by spotlighting neutrophil-associated gene expression changes in MS, a less-explored area compared to T and B cell research. By identifying pathways related to neutrophil degranulation and NET formation, we provide new perspectives on the potential roles of neutrophils in MS pathogenesis. This work fills an important gap in current literature and paves the way for functional validation of these pathways and exploration of targeted therapeutic strategies, potentially advancing treatment options for MS. Furthermore, the identification of key biomarkers such as *LCN2*, *ELANE*, *CAMP*, and *CTSG* not only enhances our understanding of MS mechanisms but also offers valuable insights for the development of diagnostic and therapeutic tools. By integrating transcriptomic analysis with functional validation, this research bridges a crucial gap between molecular discoveries and their clinical implications, thus paving the way for more effective and personalized MS therapies.

## 5 Conclusion

Our study analyzed transcriptomic data from peripheral blood neutrophils in patients with active MS, identifying significant roles for the IL-17 signaling pathway, B cell receptor signaling pathway, and NOD-like receptor signaling pathway in MS pathogenesis. The DE mRNAs in the peripheral blood neutrophils of MS patients may contribute to disease mechanisms through their regulation of neutrophil degranulation and the formation of NETs. Importantly, *LCN2*, *LTF*, *ELANE*, *CAMP*, and *CTSG* emerge as potential biomarkers although their roles in MS progression warrant further validation. These findings enhance our understanding of neutrophil-associated pathways in MS and provide a basis for future diagnostic and therapeutic advancements.

## Data Availability

The data supporting the findings of this study have been deposited in the Gene Expression Omnibus (GEO) database under accession number GSE288904. The dataset can be accessed via the following link: https://www.ncbi.nlm.nih.gov/geo/query/acc.cgi?acc=GSE288904.

## Footnotes

^1^ https://cloud.oebiotech.com/task/detail/array-enrichment/

^2^ https://string-db.org/
