# Supplementary figures and images for: Alterations in neutrophil mRNA profiles in multiple sclerosis and identification of candidate genes for further investigation
Source: Front Neurol. 2025 Feb 17;16:1548196. doi: 10.3389/fneur.2025.1548196 (PMC11873095; doi:10.3389/fneur.2025.1548196)

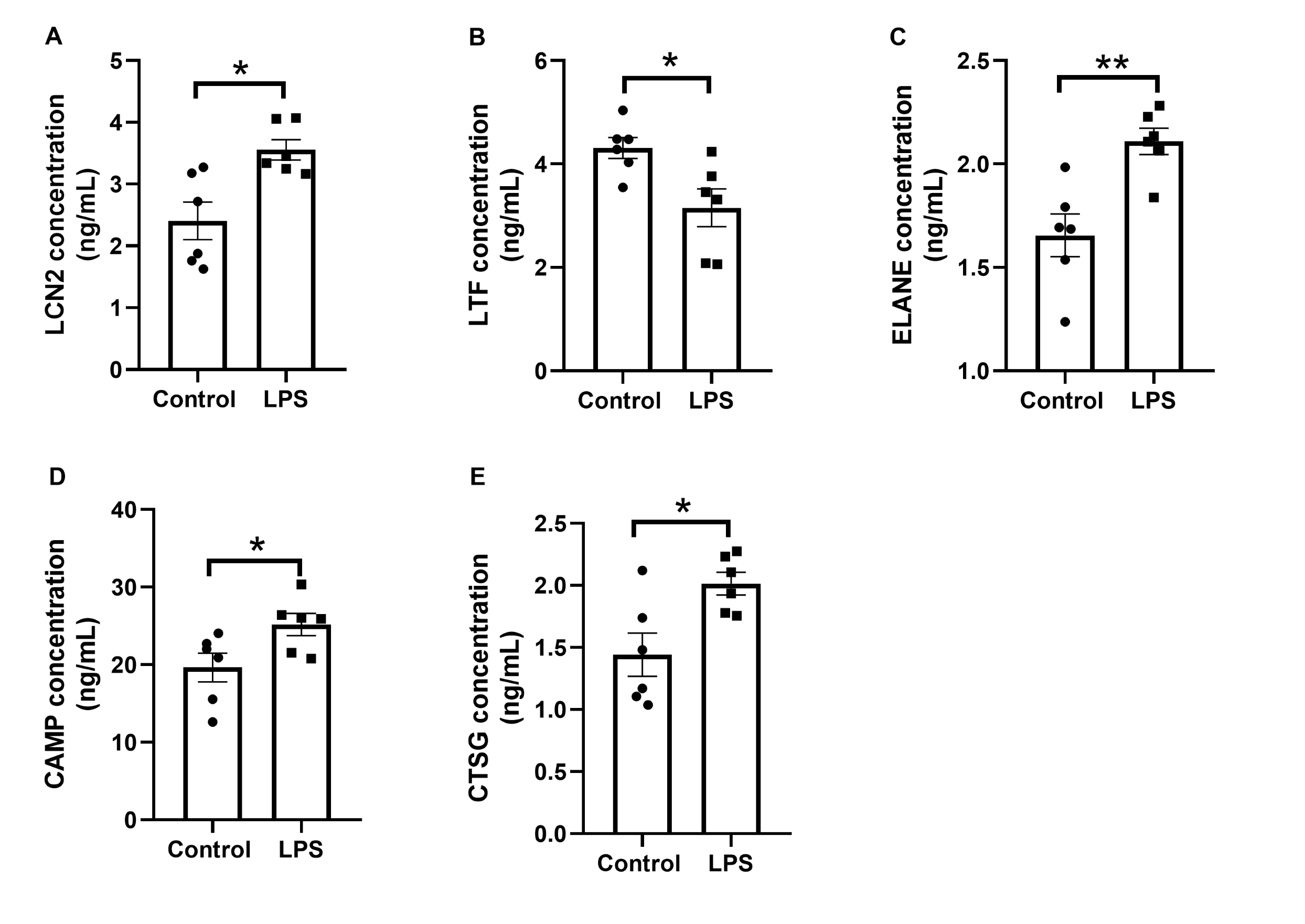

Supplement: Supplementary file 1 [file Image_1.tif]
