# Supplementary material for: Alterations in neutrophil mRNA profiles in multiple sclerosis and identification of candidate genes for further investigation
Source: Front Neurol. 2025 Feb 17;16:1548196. doi: 10.3389/fneur.2025.1548196 (PMC11873095; doi:10.3389/fneur.2025.1548196)
Supplement: Supplementary file 2 [file Table_1.docx]

**Supplementary Figure 1**

**Title:** Protein Expression Analysis of LCN2, LTF, ELANE, CAMP, and CTSG in Cell Culture Supernatants

**Figure Legend:**
The protein levels of LCN2, LTF, ELANE, CAMP, and CTSG in the supernatants of LPS-treated (1 μg/ml) and untreated dHL-60 cells were quantified using ELISA. (A) LCN2 protein levels were significantly elevated in the supernatants of the LPS-treated group compared to the control. (B) LTF protein levels showed a significant decrease in the LPS-treated group compared to the untreated control. (C-E) ELANE, CAMP, and CTSG levels were significantly increased following LPS treatment. Data are presented as mean ± SD, with significance determined at *P < 0.05.
